# Experiences of a ‘screen and treat’ cervical cancer prevention programme among brothel-based female sex workers in Bangladesh: A qualitative interview study

**DOI:** 10.1177/17455065211047772

**Published:** 2021-09-24

**Authors:** Emma Wilson, Sharmani Barnard, Samiya Mahmood, Olivia Nuccio, Sujit D Rathod, Raveena Chowdhury, Sabitri Sapkota, Tanzila Tabassum, Shah Halimur Rashid, Catherine Verde Hashim

**Affiliations:** 1 GOS Institute of Child Health, University College London, London, UK; 2 Public Health England, London, UK; 3 Marie Stopes Bangladesh, Dhaka, Bangladesh; 4 MSI Reproductive Choices, London, UK; 5 Faculty of Epidemiology and Population Health, London School of Hygiene and Tropical Medicine, London, UK; 6 MSI Reproductive Choices, Kathmandu, Nepal

**Keywords:** Bangladesh, cancer screening, cervical cancer, cryotherapy, sex workers, STI

## Abstract

**Objectives:**

Little is known about sex workers’ experiences of cervical cryotherapy. We sought to understand sex workers’ perspectives of ‘screen and treat’ programmes and their management of the World Health Organization post-treatment guidance to abstain from sex or use condoms consistently for 4 weeks. We explored contraceptive preferences and use of menstrual regulation services.

**Methods:**

We conducted semi-structured interviews with 16 sex workers and six brothel leaders in an urban brothel complex in Bangladesh between October and November 2018. All had undergone cryotherapy. We conducted a thematic analysis using deductive coding, informed by a priori themes, and inductive data-driven coding.

**Results:**

Most sex workers could not abstain from sex during the healing period. Consistent condom use was challenging due to economic incentives attached to condomless sex and coercive behaviours of clients. The implications of non-adherence among high-risk groups such as sex workers are not known. Use of short-acting methods of contraception was common, and discontinuation was high due to side effects and other perceived health concerns. The majority of sex workers and brothel leaders had utilized menstrual regulation services. Barriers to accessing timely menstrual regulation and other sexual and reproductive health services included limited mobility, economic costs, and discriminatory attitudes of health care workers.

**Conclusion:**

Service innovations are required to enable sex workers to abstain or use condoms consistently in the post-cryotherapy healing phase and to address sex workers’ broader sexual and reproductive health needs. Further research is required to assess the risk of HIV and other sexually transmitted infection transmission following cryotherapy among high-risk groups.

**Key Messages**

- Sex workers (SWs) appreciated the opportunity to attend a screen and treat programme for cervical cancer, although some were fearful of the procedures.
- Abstaining from sex or using condoms consistently in the 4 weeks following cryotherapy, as recommended, is a huge challenge for SWs.
- Service innovations are required to increase the acceptability of cervical cancer screening for SWs and address their broader sexual and reproductive health needs.

## Introduction

Cervical cancer is the fourth most commonly diagnosed cancer among women worldwide.^1^ The burden of disease is disproportionately distributed among women in low-resource settings. In 2018, approximately 84% of the estimated 570,000 new cases of cervical cancer and 85% of the 311,000 deaths were in low- and middle-income countries (LMICs).^2^ In Bangladesh, cervical cancer ranks second among cancer diagnoses among women, following breast cancer, with an estimated 8068 new cases and 5214 deaths recorded in 2018.^3^

The Government of Bangladesh has committed to scaling up a national opportunistic screening programme based on visual inspection of the cervix with acetic acid (VIA) to identify precancerous lesions and referral of screen-positive cases for colposcopy and treatment.^4^ Yet, as in other LMICs, coverage of VIA screening remains persistently low, compounded by challenges such as low retention of trained staff, poor linkage to onward care and treatment, and inconsistent quality standards.^5^

To expand access to screening among high-risk populations, Marie Stopes Bangladesh (MSB) offered ‘screen and treat’ VIA screening programmes to brothel-based sex workers (SWs) and urban women, including women residing in slum areas. SWs are at elevated risk of cervical cancer, given the high prevalence of human papillomavirus (HPV) among this population.^6^ VIA was utilized to identify precancerous lesions, followed by the administration of cryotherapy during the same visit to eliminate abnormal lesions among screen-positive women. In total, the programme carried out 11,296 screenings and 345 treatments between September 2015 and August 2018 (Box 1).

**Box 1.** MSI Reproductive Choices screen and treat programmes.

MSI Reproductive Choices provides screen and treat services across 18 countries in sub-Saharan Africa and Asia. Screening is conducted using visual inspection with acetic acid (VIA) and treatment for VIA-positive and eligible women with cryotherapy. Services are carried out through a variety of channels including static centres, social franchises, and outreach. MSI aims to provide a single visit approach, with cryotherapy being made available on screening sites.

World Health Organization (WHO) guidelines recommend that clinicians counsel screen-positive women on abstaining from vaginal intercourse for 4 weeks following treatment or consistent condom use if abstinence is not feasible.^7^ These strategies enable the cervix to heal after cryotherapy and reduces the risk of acquiring or transmitting HIV and other sexually transmitted infections (STIs). MSB clients were also counselled on safe sex and family planning, offered syndromic STI management, and referred to HPV vaccination services if aged 26 years or younger.

Cervical cryotherapy has high acceptability in diverse settings,^8,9^ yet not all women are able to adhere to the post-treatment guidelines.^8^ A literature review identified five studies that explored women’s sexual practices following cryotherapy.^9^ Although most women adhered to the guidelines, two studies from Kenya and South Africa found that 22.7% (5/22) and 23.8% (226/949) of women, respectively, did not abstain or use condoms consistently in the post-treatment healing phase. In some contexts, women reported coercion or pressure to have sex (including from husbands), or struggled to obtain condoms.^10^ To our knowledge, no studies have explored how SWs manage or adapt sexual practices following cryotherapy.

We conducted a qualitative study with brothel-based SWs and brothel leaders who received cryotherapy from the MSB screening programme. Our aim was to understand SWs’ experiences of the screening programme and gain insights into their management of the post-treatment healing phase. We also sought to understand SWs’ contraceptive preferences and their use of menstrual regulation (MR) services (Box 2) to improve delivery of sexual and reproductive health (SRH) services to this population.

**Box 2.** Legal framework for sex work and MR in Bangladesh.

*Sex work* It is estimated that there are approximately 100,000 sex workers in Bangladesh, the majority of whom operate from hotels or in street settings.^11^ Under the Oppression of Women and Children (Special Enactment) Act, 1995, it is illegal for third parties to import, export, sell, or hire women for sex work. Public solicitation is also prohibited. However, sex work in private is legal.^11^ There is also a quasi-legal system of brothels, which are monitored by local authorities. This brothel system comprised 14 official brothel complexes across the country, which house approximately 4000 sex workers. Brothel-based sex workers must pay a fee to the police and obtain a certificate from the magistrate to operate in the brothel.^11^ However, it is understood that bonded labour and exploitation are prevalent within brothels.^12^ *Menstrual Regulation* Induced abortion is illegal in Bangladesh except to save a woman’s life. However, menstrual regulation (MR) in order to ‘regulate the menstrual cycle when menstruation is absent for a short duration’^13^ is legal and has been part of the Bangladesh Family Planning programme since 1979. Permitted MR procedures include manual vacuum aspiration (MVA) and menstrual regulation with medication (MRM), using a combined regimen of mifepristone and misoprostol. MVA is available free of charge in public sector facilities and is permitted up to 10–12 weeks after a woman’s last menstrual period, while MRM is approved for use in public facilities up to 9 weeks after a woman’s last menstrual period or it can be acquired on prescription at pharmacies.^14–16^ It is estimated that there were 1,194,000 induced abortions in 2014 (29 per 1000 women aged 15–49 years), and 257,000 women were treated for complications resulting from induced abortions.^15^

## Methods

We used a qualitative study design to explore SWs’ experiences of a screen and treat cervical cancer screening programme and to gain insights into their broader SRH behaviours. Between 22 October and 19 November 2018, we recruited 16 SWs and six leaders (who had also worked as SWs) from one urban brothel complex in Bangladesh that houses approximately 1000 SWs. MSB held three screen and treat clinics at the brothel complex in 2017. Study participants (SWs and brothel leaders) were identified from MSB client lists and approached via telephone call. Eligibility for the study was based on having received cryotherapy from MSB in the previous 18 months and being at least 18 years of age. Participants were purposively selected to capture a range of experiences based on age, relationship status, and position within the brothel hierarchy. Of the 82 women who had undergone cryotherapy in 2017, 22 women (16 SWs and 6 brothel leaders) were identified, and all agreed to take part.

Semi-structured individual interviews were carried out in Bengali by two trained female research assistants (RAs). The RAs were selected from a cadre of fieldworkers who had worked for MSB’s Shokhi project, delivering free SRH and legal services to women living in 15 slums in Dhaka. The RAs had prior experience of conducting qualitative research and they received 3 days of additional training on qualitative interviewing techniques, study procedures, and research ethics. The RAs informed participants that they worked for MSB and they were interested to learn of their experiences of cryotherapy in order to improve services. Data collection was overseen by a research manager (T.T.). All participants provided written informed consent, and interviews were conducted in a private space within the brothel complex. Interviews were audio-recorded and lasted approximately 1 hour and followed a topic guide which covered health and relationships, experiences of the MSB screen and treat programmes, understanding and adherence to post-treatment guidance, contraceptive preferences, and MR seeking behaviours (see Supplementray File 1). The topic guide was piloted with two SWs prior to initiation of data collection. The RAs recorded fieldnotes during and after each interview using Microsoft Word. All participants were offered referrals to legal and other health services as needed (see Box 2 for contextual information on the legal frameworks governing sex work and MR in Bangladesh).

Interviews were transcribed verbatim. Transcripts were translated into English by two professional translators. Two researchers (E.W. and C.V.H.) conducted a thematic content analysis,^17^ using both deductive coding, informed by a priori themes, and inductive data-driven coding to enable the identification of new themes.^18^ The coding framework was developed iteratively and collaboratively. Data were coded and managed using Microsoft Excel. All queries related to context, interpretation, and analytical conclusions were discussed with the local MSB team.

Ethical approval was obtained from MSI Reproductive Choices’ Ethics Review Committee (Ref. No. MSI ERC 004-18) and the Bangladesh Medical Research Council (Ref. No. BMRC/NREC/2016-2019/734).

## Findings

### Profile of participants

The 16 SWs were aged between 20 and 55 years (median, 26.5 years). Most had received no formal education. Seven were in an informal union and 10 had at least one child (Table 1).

**Table 1.** Participant characteristics (sex workers only).^a^

	Sex workers (*n* = 16)	
	*n*	%
Age group (years)		
20–29	10	62.5
30–39	3	18.8
40+	3	18.8
Median age (years) (IQR)	26.5 (25–32.5)	
Education		
None	12	75.0
Incomplete primary	1	6.3
Complete primary	3	18.8
Marital status		
Unmarried	6	37.5
Married or informal union	7	43.8
Polygamous marriage	0	0.0
Divorced	1	6.3
Separated	2	12.5
No. of children		
None	6	37.5
1	7	43.8
2	2	12.5
3	1	6.3

IQR: interquartile range.

Percentages may not add up to 100 due to rounding.

a Detailed demographic information of brothel leaders (*n* = 6) is not presented to preserve participant anonymity.

Of the six brothel leaders, one was currently active in sex work and the remainder had been SWs previously. Their median age was 39 years and all had children. Four leaders were in an informal union and most had no education.

### Knowledge and beliefs surrounding cervical cancer

Cervical cancer was widely understood to be a serious condition, which could potentially lead to death. While HPV was not mentioned as a cause of cervical cancer, many SWs (*n* = 11) described a link between cervical cancer and STIs or unprotected sex more broadly.

Having sex while the cervix is ‘wet’, due to discharge or menstrual blood, was also perceived to be risky, as a ‘wet’ cervix is considered to be weak and easily damaged:

It attacks for having sex during menstruation. In this time uterus [cervix] remains sensitive [. . .] Then it turns into red colour and pus discharges from blisters, and then cervical cancer emerges from here. (SW, aged 20–29 years)

Staying ‘clean’ and washing after sex were considered important preventive measures. Four SWs suggested that MR, particularly surgical methods, could increase women’s risk of cervical cancer.

### Experience of the MSB screening programme

The opportunity to learn of their cancer ‘status’, receive free treatment, and potentially resolve ongoing symptoms including discharge, vaginal itching, and abdominal pain were cited as key motivating factors for attending the MSB screen and treat programme.

Many SWs described feeling frightened of the medical equipment and some had understood that the screening would involve the removal of their uterus. One SW felt embarrassed to remove her clothes, while in several accounts, cryotherapy was reported to be physically painful:

When they dragged it then I thought that what are they dragging? It hurts me a lot that I am dying. After that still some days I felt pain . . . (SW, aged 20–29 years)

However most SWs described feeling reassured by staff and reported high levels of trust in MSB personnel, who ‘spoke nicely’ and showed them ‘respect’ and ‘affection’.

### Abstinence from sex and sex work

Most SWs had difficulty recalling the post-treatment abstinence and condom use guidelines, and the majority (*n* = 14) recounted engaging in sex work within a few days of receiving cervical cryotherapy. Sex work is clearly a necessity for daily survival, enabling SWs to pay their room rent and meet their financial commitments to their children and families:

Here we live from hand to mouth. If there is no job, salary is not given, to provide house rent as well as not to take food . . . We are bound to have sex. (SW, aged 20–29 years)

So if I remain at rest for one month . . . The landlord will come after a while and say, ‘Pay me the rent’ . . . I am compelled to have sex, since beside this I do not have any other work. (SW, aged 20–29 years)

Abstinence was viewed as a potential risk to future earnings, as clients may seek services elsewhere:

If I ask the man who has come to have sex with me to go away today because I could not entertain him, [. . .] So, if that man does not come up then I have lost a client [. . .] (SW, aged 20–29 years)

Therefore, only ‘solvent’ people with sufficient wealth or familial support were perceived to be in a position to comply with the guidance, including brothel leaders in possession of indentured SWs. Nevertheless, two SWs reported abstaining for the full month. One SW used her savings, while the second sent her lover (babu) to his hometown and had no clients during this time. Babus are regular clients with whom SWs have an emotional attachment. Some SWs may refer to their babu as their ‘husband’. In some cases, SWs do not charge their babus for sex.

### Condom negotiation

Among the SWs who resumed sexual activity, a mixed picture emerged of condom use. Some SWs (*n* = 7) reported using condoms for all, or part of, the post-treatment phase, and they suggested that condoms are consistently negotiated with male clients. Other accounts were less clear, and many SWs described limited bargaining power, particularly when clients offer economic incentives for condomless sex:

I see that most of the people don’t want to use a condom. There are 2 to 3 people at a place. One of them offers 500 taka without a condom. If I miss the offer, then I might earn 300 taka. Then it will be a loss for me. (SW, aged 30–39 years)

Four SWs described scenarios where they were subjected to violent treatment by clients. Women’s agency in sexual decision-making appeared to be further mediated by age and position within the brothel hierarchy. Younger, indentured, SWs can be forced to have condomless sex to maximize the earnings of brothel leaders, while an older SW described engaging in higher risk sexual practices to increase demand for her services.

While many SWs expressed a normative desire to use condoms with clients, this was not an option with babus (lovers) or husbands, as condoms are symbolic of commercial rather than romantic encounters:

[. . .] I don’t treat him as a client. He is my husband, my love. I don’t take money from him. So I don’t use condom with him. (SW, aged 20–29 years)

### Contraceptive preferences

Aside from condom use, SWs reported use of other short-acting methods, such as the contraceptive pill, and to a lesser extent, injectables. Yet discontinuation or inconsistent use was common due to health concerns or unpleasant side effects. Weight gain, dizziness, nausea, and menstrual problems were linked to the contraceptive pill, while the cessation of menstruation was a common concern associated with injectables:

If I use pill, I will be fatty, even I don’t want. When anybody uses pill suddenly she will be fatty and when somebody left it then it feels bad to our body, also head spins [. . .] [SW, aged 20–29 years]

Difficulties acquiring regular supplies of short-acting methods, and remembering to take the contraceptive pill, were also mentioned as potential barriers to continued short-term method use.

According to one brothel leader, the intrauterine device (IUD) is not commonly used in the brothel, as it is ‘forbidden’ due to the perceived risk of perforating a woman’s uterus. None of the SWs mentioned the use of implants.

### MR

The majority of SWs (11/16) and leaders (5/6) reported having MR at least once in their lifetime, while five SWs and five leaders reported having MR more than once. SWs accessed both medical and surgical MR in clinics, non-governmental organization (NGO) clinics, and public hospitals; and medical MR was also obtained from informal providers such as pharmacies.

SWs described significant barriers accessing formal health services due to cost and the discriminatory attitudes of health care workers (HCWs). Health professionals may deny MR services to SWs, forcing women to lie about their personal circumstances and give false addresses in order to gain access to services:

In case of outside doctor whom we need to pay [. . .] we cannot introduce us to them as sex worker and came from brothel. This cannot be said, and we feel shy. . . (Sex worker, aged 20–29 years)

Most participants were aware of menstrual regulation with medication (MRM), which was perceived to be convenient, particularly for those who struggle to access formal providers. MRM was also understood to be effective in early pregnancy, while surgical methods would be required for MR at later gestation.

Nevertheless, five participants reported complications following MRM (Note it is not known whether MRM was self-administered or provider-administered.), including incomplete MR or failed MR that required follow-up care in health facilities. From four accounts, it was possible to discern that participants had undergone MR beyond the legal time period. In one harrowing account, an SW described having MRM at 5 months within the brothel:

I had aborted that by taking drug. [. . .] As it was not successful there, they gave me medicine for taking it three times. But I took all the three at one time [. . .] After I had taken the medicine, it was aborted at night when no one was awake, everyone was asleep. It was 2 am at night [. . .] But later on it was felt, there was severe pain . . . (Sex worker, aged 20–29 years)

### Attitudes of the Sardarnis (brothel leaders)

Leaders were keen to position themselves as sympathetic to the needs of the SWs in their charge, emphasizing efforts to protect SWs from abusive clients, support condom use, and facilitate timely access to health services. One leader did, however, admit that she stopped an SW from attending the cervical cancer screening programme to avoid loss of earnings, while another suggested leaders feared that MSB staff might report them to the authorities.

Leaders also recognized that SWs would have limited possibilities to abstain from sex work, ‘as the girls have to earn money on daily basis’, and leaders themselves would also struggle to survive:

If there is no income, then how would I eat? [. . .] I have only one girl, if I don’t have her, then how would I lead life? (Brothel leader)

One leader was explicit in her belief that sex workers are mere commodities:

I have bought her with money. The girl would give me money by having sex with the people. (Brothel leader)

## Discussion

The SWs interviewed for this study valued the opportunity to attend the MSB screen and treat programme. Access to free VIA screening, the provision of immediate treatment for screen-positive women, and respectful attitudes of HCWs were cited as particular advantages of the programme. However, a number of women had limited knowledge of VIA screening and were fearful of the procedures and equipment.

During the post-treatment phase, abstinence was simply not feasible due to participants’ dependence on sex work for their day-to-day survival. Brothel-based SWs in Bangladesh are charged exorbitant rents,^11^ and meeting this daily fee was cited as a particular source of anxiety in this study.

Consistent condom use was an ongoing challenge for many, due to the economic incentives attached to condomless sex as well as the violent or coercive behaviours of some clients within sexual negotiations. The use of condoms with *babus* was not common, echoing studies that have found that condomless sex is often used to establish intimacy within non-commercial relationships and to create symbolic distance from sex work.^19^

It is evident that SWs can face significant barriers in adhering to the post-treatment guidelines, due to their constrained social and economic agency. The implications of this are unknown. Current international guidelines are based on the premise that the benefits of cryotherapy outweigh any potential risks of infection.^20^ Yet the evidence on HIV or STI transmission following cryotherapy is scant.^20,21^ One randomized controlled trial (RCT) conducted in South Africa found no evidence of increased rates of HIV seroconversion among women screened and treated via cryotherapy compared to a control group who were screened and assigned to delayed evaluation.^22^ However, the study lacked statistical power to detect potentially small differences in seroconversion rates, and findings may not be generalizable to SWs for whom protective measures such as abstinence and consistent condom use are particularly challenging.^21,23^ Further research is needed to estimate any excess risk of HIV transmission and other STIs following cryotherapy particularly among key populations including SWs and their clients. Such insights may inform revisions to the cryotherapy guidelines – for example, consensus on the optimal duration of post-treatment abstinence to balance both health risks and service user constraints, as well as specific recommendations for providers, such as the provision of economic incentives to aid compliance.

In line with other research with SWs in Bangladesh, we found a high unmet need for effective contraceptive methods. Most women reported at least one unintended pregnancy and most SWs had used MR services at least once. Four SWs indicated that they had sought terminations beyond the legal time limit for MR. This is unsurprising given the significant barriers faced by SWs in accessing timely MR and other SRH services, due to highly discriminatory and stigmatizing attitudes of HCWs as well as inconvenience, time, and cost constraints.^24,25^

Furthermore, in some accounts, SWs described complications following MRM, such as incomplete or failed MR. While it was not clear where participants had acquired MRM, it is critical that quality standards are upheld across all MRM providers, including correct advice on dosage, contraindications, and recommended follow-up.^26,27^

While none of the SWs in our study were debt-bonded at the time of interview, the accounts of brothel leaders and SWs indicated that bonded SWs are subject to extreme exploitation and have limited agency to negotiate safe sexual practices and secure timely access to health services. These women are, therefore, particularly vulnerable to a range of SRH concerns, including STIs, unwanted pregnancy, and unsafe abortions.

Our study had several strengths. Few studies have explored SWs’ and brothel leaders’ experiences of a cervical cancer prevention programmes. Interviews were conducted in the local language by female research assistants, who were experienced in conducting research on sensitive topics and had previously worked with marginalized women in Dhaka. One analyst (C.V.H.) attended the data collection phase and was able to apply this contextual understanding to the analyses and interpretation.

The main limitation is potential recall error as interviews were conducted between 12 and 18 months after participants received cervical cryotherapy due to delays in securing ethical approvals. It was not possible to recruit women who had undergone recent cryotherapy as the MSB screen and treat programme was funded for a limited period and had ceased operations by the time ethical approvals were granted. Nevertheless, this time lag is unlikely to have affected our broader understanding of sexual decision-making, condom negotiation, and economic dependence on sex work in this context. Participants were recruited from a single urban brothel complex, and therefore wider generalizability beyond this context may be limited. Furthermore, the experiences of participants who agreed to be followed up and interviewed may not be representative of all MSB SW clients. Data saturation (when new data broadly repeat previously collected data^28^) was achieved for most of our research themes. However, we cannot be certain that saturation was reached with respect to the data on adherence to the post-cryotherapy guidelines. Only two SWs in our sample reported abstaining from sex in the healing phase, and due to time constraints, we were unable to explore the possibility of identifying additional participants who may have also abstained, to ensure we reached saturation within this category.

Responses were inevitably shaped by the interactional context of the interview and the professional identity of the interviewers, who were former MSB outreach workers. Therefore, some participants may have positioned themselves as more concerned with SRH matters than was the case. Participants may also have been reluctant to report stigmatized practices such as polygamy.

## Conclusions and recommendations

Our findings underline the importance of sensitizing women on screening procedures, to allay fears and reinforce post-treatment recommendations. This could be achieved via community outreach and education alongside renewed focus on pre- and post-treatment counselling. Counselling should also extend to male sexual partners (e.g. babus) and brothel leaders in order to garner their support. Emphasis should be placed on condom use and condom negotiation strategies with SWs, their clients, and sexual partners, as abstinence is unlikely to be feasible.

Service needs extend beyond those of cancer screening in this context. SWs should be involved in the co-development of comprehensive, non-judgemental SRH services, including quality MR and post-MR care. Critically, efforts are needed to address discriminatory attitudes towards SWs and improve quality standards among SRH providers, particularly those providing MR services, while strengthening referral networks to legal and other forms of social support.

Further research is required to assess the risk of HIV and other STI transmission following cryotherapy among high-risk groups. Such evidence may prompt new provider recommendations such as the provision of economic incentives to facilitate safe sex practices among marginalized groups.

## Supplemental Material

sj-docx-1-whe-10.1177_17455065211047772 – Supplemental material for Experiences of a ‘screen and treat’ cervical cancer prevention programme among brothel-based female sex workers in Bangladesh: A qualitative interview study Click here for additional data file. Supplemental material, sj-docx-1-whe-10.1177_17455065211047772 for Experiences of a ‘screen and treat’ cervical cancer prevention programme among brothel-based female sex workers in Bangladesh: A qualitative interview study by Emma Wilson, Sharmani Barnard, Samiya Mahmood, Olivia Nuccio, Sujit D Rathod, Raveena Chowdhury, Sabitri Sapkota, Tanzila Tabassum, Shah Halimur Rashid and Catherine Verde Hashim in Women’s Health
