# Supplementary material for: Experiences of a ‘screen and treat’ cervical cancer prevention programme among brothel-based female sex workers in Bangladesh: A qualitative interview study
Source: Womens Health (Lond). 2021 Sep 24;17:17455065211047772. doi: 10.1177/17455065211047772 (PMC8477689; doi:10.1177/17455065211047772)
Supplement: sj-docx-1-whe-10.1177_17455065211047772 – Supplemental material for Experiences of a ‘screen and treat’ cervical cancer prevention programme among brothel-based female sex workers in Bangladesh: A qualitative interview study [file sj-docx-1-whe-10.1177_17455065211047772.docx]

## Interview topic guide- sex worker

**Background Information**

*Interviewer please complete:*

|  | **Details** |
| --- | --- |
| Interview date: |  |
| Interview place: |  |
| Interview start time |  |
| Interview end time: |  |
| Respondent ID number: |  |
| Age: |  |
| Marital status: |  |
| Main job/activity: |  |
| Education level (tick one): | …… No formal education  .....Primary (incomplete)  .....Completed primary  .....Secondary (incomplete)  .....Completed secondary  .....Higher education |
| Number of living children: |  |
|  | **Details** |

1. **Ice breakers**
   1. How long have you been doing this work?
   2. What do you like about the work?
   3. What do you dislike about the work?
   4. Have you worked in this facility for long?
      1. Where did you work before?
      2. What was different about it?
   5. Have you done any other types of work?
   6. Are you married?
      1. How long have you/were you married?
      2. Do you have any children?
2. **Health and relationships**
   1. What do you think about the number of hours women in this facility work?
      1. When women are working do they have much time to interact with colleagues?
      2. How many men do women typically see per day?
   2. What do women think of the working conditions here?
      1. What would they change?
      2. What would they keep the same?
   3. Have you seen many health campaigns visit the facility?
      1. What types of campaigns?
      2. What did you think of them? Why?
      3. Have you accessed any of them? Why/why not?
   4. Have you used family planning in the past?
      1. What did you like/dislike about it?
   5. What experiences have you had with menstrual regulation (MR)?
      1. How difficult or easy is it for women to access MR here?
   6. Can you describe how often you see a health care provider?
      1. What sort of conditions would you seek help for?
      2. Do you feel comfortable seeking health care?
   7. Can you describe how you manage your menstrual hygiene?
3. **Cervical cancer**

Next, I would like to ask you about cervical cancer

- 1. What do you know about cervical cancer?
  2. Where did you learn about cervical cancer?
  3. Do you think the disease is a concern for sex workers?
     1. Why?
  4. How do you think the other women working here feel about CC?
     1. What makes you think that?
  5. How do you think the brothel leaders working here feel about CC?
     1. What makes you think that?
  6. How does your husband/family feel about CC?

1. **CCS and cryotherapy**
   1. Can you describe what happened the day MSB came to do CCS and cryo here?
      1. What made you get tested?
      2. How did you feel that day?
   2. Did you talk to any MSB staff prior to the CCS procedure?
      1. What did they talk to you about?
      2. How did it make you feel?
      3. What did you know about what might happen if you tested VIA +ve?
   3. What happened after the procedure?
      1. Did anyone speak to you after the procedure?
      2. What did they tell you?
      3. Were you given any information? If so, what was it?
      4. Did you understand the information that you were given?
   4. Imagine you are the doctor and I am the patient, can you explain what I should and shouldn’t be doing if I have just had cryotherapy?
   5. What do you think about screening in the future?
      1. Would you do it?
      2. When would you do it?
         1. Can you explain why?
2. **Adherence to guidelines**

Next, I’d like to ask you some questions about how you felt about the guidelines that you were told to follow after receiving cryotherapy.

1. What do you think of these guidelines?
2. What do you think of having to abstain from sexual intercourse for four weeks?
   1. Why did you feel that way?
   2. Would anything stop someone from being able to follow these guidelines, such as:
      1. Anything/anyone at work?
      2. Anything/anyone at home?
      3. What about any financial issues?
   3. Were you able to follow these guidelines?
      1. If not, can you tell me why not?
      2. What could have helped you or did help you to follow these guidelines?
         1. Is there anything that MSB did/could have done?
         2. Is there anything that your husband did/could have done?
         3. Is there anything that your boss/leader did/could have done?
         4. Is there anything that your friends did/could have done?
   4. Do you know of other women’s experiences following these guidelines?
      1. What do you think stopped them?
      2. What do you think could have helped them?
3. **Overall experience**

Now I’m going to ask you some questions about your experiences (both good and bad) that you may have had as a result of the CCS-cryotherapy

- 1. Did you have any experiences (please mention both good and bad) that you think were a result of the cryo-therapy
     1. Symptoms/ recovery?
     2. Trying to adhere to the guidance?
     3. Not adhering to the guidance?
  2. Did you experience any financial loss/gain as a result of the screening?
     1. If not for you- do you know of anyone who experienced financial loss/gain as a result of screening?
     2. Can you describe why this would happen
  3. Did the screening have any negative/positive impact on your work life?
     1. Can you describe this?
  4. Did the screening have a negative/positive impact on your home life?
     1. Can you describe this?

1. **Family Planning**
   1. Were you using a FP method on the day that the CCS camp came?
      1. If so, which one?
      2. Why were you using that one?
   2. Did MSB staff talk to you about FP methods on the day?
      1. Which FP methods did they discuss?
      2. Did the visit from MSB change any feelings you had about FP?
      3. If so, what changed and what made your feeling change?
   3. Have you changed your FP methods since the visit?
      1. If so, why? which services?
      2. If not, why not?

We are getting to the end of the interview. Do you have any comments to make about cervical cancer screening, family planning provision or anything else?

1. **Closing statement**

Thank you for participating in this interview. Information that you have given me will help Marie Stopes to make contraception and cervical cancer screening services better quality. This is the end of the interview; thanks a lot for agreeing to participate today.

## Interview topic guide- brothel leader

**Background Information**

*Interviewer please complete:*

|  | **Details** |
| --- | --- |
| Interview date: |  |
| Interview place: |  |
| Interview start time |  |
| Interview end time: |  |
| Respondent ID number: |  |
| Age: |  |
| Marital status: |  |
| Main job/activity: |  |
| Number of years in job: |  |
| Education level (tick one): | ---no formal education  .....Primary (incomplete)  .....Completed primary  .....Secondary (incomplete)  .....Completed secondary  .....Higher education |

1. **Ice breakers**
2. How long have you been working here?
3. What do you like about working here?
4. What do you dislike about working here?
5. Have you worked in this facility for long?
6. Where did you work before?
7. What was different about it?
8. Have you done any other types of work?
9. Are you married?
10. How long have you/were you married?
11. Do you have any children?
12. **Health and relationships**
13. What do you think of the working conditions for leaders in this facility?
    1. Are you under any pressure to achieve targets?
       1. Can you describe where this pressure comes from?
14. What do you think about the number of hours women in this facility work?
15. When women are working do they have much time to interact with colleagues?
16. How many men do women typically see per day?
17. What do you think women think of the working conditions here?
18. What would they change to make it better?
19. What would they keep the same?
20. What sort of changes do you think should be made to this facility?
21. Have you seen many health campaigns visit the facility?
22. What types of campaigns?
23. What did you think of the campaigns? Why?
24. Do most of the women use family planning? If so, what sort?
25. Do you have an opinion on what FP method is best for the women working here? What is it? Why?
26. What experiences have you had with menstrual regulation (MR)?
27. How easy or difficult is it for women to access MR here?
28. What do you think about women seeking health care?
29. What sort of conditions should they seek help for?
30. Where should they seek it from?
31. **Cervical cancer**

Next, I would like to ask you about cervical cancer

1. What do you know about cervical cancer?
2. Where did you learn about cervical cancer?
3. Do you think the disease is a concern for sex workers?
4. Why?
5. How do you think the other women working here feel about CC?
6. What makes you think that?
7. How do you think the brothel leaders working here feel about CC?
8. What makes you think that?
9. What influences women and brothel leaders feeling about CC?
10. **CCS and cryotherapy**
11. Can you describe what happened the day MSB came to do CCS and cryo here?
12. Did you encourage the women to get tested?
13. Is there any reason why you would discourage women from being tested?
14. Have you heard of any leaders that do that?
15. Did you talk to any MSB staff prior to the CCS procedure?
16. What did they talk to you about?
17. Did you have any worries, if so, what where they?
18. What did you know about what might happen if women in your care tested VIA +ve?
19. What happened after the procedure?
20. Did anyone from MSB speak with you after your women had the procedure?
21. What did they tell you?
22. Were you given any information? If so, what was it?
23. Did you understand the information that you were given?
24. Imagine you are the doctor and I am the patient, can you explain what I should and shouldn’t be doing if I have just had cryotherapy?
25. What do you think about screening in the future?
    - 1. Do you want it to happen?
      2. Would you let it happen?
         1. Can you explain why?
26. **Adherence to guidelines**

Next, I’d like to ask you some questions about how you felt about the guidelines that the women were told to follow after receiving cryotherapy.

1. What do you think of these guidelines?
2. What do you think of them having to abstain from sexual intercourse for four weeks?
   1. Why did you feel that way?
   2. Would anything stop a woman from being able to follow these guidelines, such as:
      1. Anything/anyone at work?
      2. Anything/anyone at home?
      3. What about any financial issues?
   3. Did you stop or encourage any of your women from following these guidelines?
      1. If so, can you tell me why?
      2. What could have helped/did help you to allow women to follow these guidelines?
         1. Is there anything that MSB did/could have done?
         2. Is there anything that the women did/could have done?
         3. Is there anything that your boss/leader did/could have done?
   4. Do you know of anyone (else)’s experience following these guidelines?
      1. If anything stopped them, what do you think it was?
      2. If anything helped them, what do you think it was?
      3. What do you think could have helped them?
3. **Overall experience**

Now I’m going to ask you some questions about your experiences (both good and bad) that you may have had as a result of the CCS-cryotherapy

1. Did you hear of any experiences (please mention good and bad) that you think were a result of the cryo-therapy
   - 1. Symptoms/ recovery?
     2. Trying to adhere to the guidance?
     3. Not adhering to the guidance?
     4. Financial loss/gain?
        1. Can you explain why?
2. **Family Planning**
3. Did MSB staff talk to you about FP methods for the women on the day?
4. Which FP methods did they discuss?
5. Did the visit from MSB change any feelings you had about FP?
6. If so, what changed and what made your feeling change?
7. Have you changed your FP methods since the visit?
8. If so, why? which services?
9. If not, why not?

We are getting to the end of the interview. Do you have any comments to make about cervical cancer screening, family planning provision or anything else?

1. **Reminder of importance of post-cryotherapy abstinence**

Thank you for your time today. Before we conclude, I would like to provide you with some materials highlighting: the importance of cervical screening, timing and frequency of screening, prevention of cervical cancer, importance of abstinence after cryotherapy to prevent risk of infection, and recommended abstinence duration. Do you have any questions on the information provided in these materials?

1. **Closing statement**

Thank you for participating in this interview. Information that you have given me will help Marie Stopes to make contraception and cervical cancer screening services better quality. This is the end of the interview; thanks a lot for agreeing to participate today.
